# Evaluation of screw pull-out from plate fixation of en bloc distal radius resection with ulnar reconstruction: Finite element analysis and comparison with experiments on Thiel cadavers

**DOI:** 10.1063/5.0248553

**Published:** 2025-06-11

**Authors:** Wares Chancharoen, Theingi Nwe, Saran Seehanam, Napawan Taradolpisut, Thewarid Berkband, Thanapon Chobpenthai, Chavin Jongwannasiri, Laphatrada Yurasakpong

**Affiliations:** 1 Laboratory of Artificial Intelligence and Innovation in Medicine (AIIM), Princess Srisavangavadhana Faculty of Medicine, Chulabhorn Royal Academy, 906 Kamphaeng Phet 6 Road, Talat Bang Khen, Lak Si, Bangkok 10210, Thailand; 2 Department of Mechanical Engineering, Naypyitaw State Polytechnic University, Thirimandhain Junction, Pubbathiri Township, Naypyitaw 15014, Myanmar; 3 Department of Anatomy, Faculty of Science, Mahidol University, 272 Rama VI Road, Ratchathewi, Bangkok 10400, Thailand; 4 Department of Anatomy, Faculty of Medicine, Khon Kaen University, 123 Moo 16, Mittraphap Road, T. Naimuang A. Muang, Khon Kaen 40002, Thailand

## Abstract

Fractures of the distal radius often require surgical intervention, with plate fixation being a standard stabilization method. Screw loosening and pull-out propose significant complications, necessitating comprehensive understanding of fixation stability factors. This study introduces a novel approach by the combination of finite element analysis (FEA) and experimental investigations on Thiel cadavers to evaluate screw pull-out behavior from plate fixation in en bloc distal radius resection with ulnar reconstruction. In comparison with previous investigations that used computational modeling or fresh-frozen cadaveric specimens, in the present research, FEA predictions specifically experimentally confirm the usage of Thiel cadavers, which better preserve soft tissue elasticity and hydration, thus more closely reflect *in vivo* conditions. Experimental set-up consisted of bending tests on cadavers and screw pull-out tests in Thiel-cadaveric radius specimens mimicking physiological conditions that induce the effects of screw pull-out. Finite element analysis and simulation were conducted using realistic clinical cases. Biomechanical test results indicated locking-plate deformation and screw loosening, particularly at locations closest to the ulnar bone gap. Torque measurements established various degrees of screw loosening, with the screws closest to the bone gap indicating maximum loosening. FEA demonstrated critical distributions of stresses in screws and locking plates, with good correlations to experimental findings. Screw pull-out force analysis showed vulnerability to loosening, particularly in the area of bone gaps, with findings consistent between biomechanical testing and FEA. This study offers valuable information on the surgical implications and biomechanical considerations of plate fixation for en bloc distal radius resection with ulnar reconstruction.

## I. INTRODUCTION

Fractures of the distal radius often necessitate surgical intervention, with plate fixation serving as a standard method for stabilizing the fracture site. However, in cases of severe injury or tumor resection requiring en bloc distal radius resection with ulnar reconstruction, the biomechanical challenges associated with plate fixation become significantly amplified. Giant cell tumors necessitate extensive resection of the bone, often leading to en bloc distal radius resection with ulnar translocation, a procedure acknowledged for its complexity and challenges for surgeons.^1,2^ A significant issue with these treatments is the possibility of screw loosening and pull-out, which might risk surgical results and fixation stability. Therefore, understanding the biomechanical aspects, including screw loosening and pull-out, is paramount in optimizing surgical outcomes and improving patient recovery. The evaluation of screw pull-out from plate fixation of en bloc distal radius resection with ulnar reconstruction has significant clinical implications. While finite element analysis (FEA) has been widely used to model and predict the mechanical behavior of orthopedic implants, there has been limited validation of these simulations using real bone specimens under physiologically relevant conditions.

Thiel cadavers offer a realistic platform for biomechanical simulations by providing a unique advantage as they preserve soft tissue elasticity, hydration, and mechanical integrity, closely mimicking *in vivo* conditions. Thiel embalming, with its crucial chemical component, ammonium nitrate, plays a pivotal role in preserving the lifelike color of tissues, enhancing their realism.^3^ The Thiel embalming process itself ensures that cadavers remain flexible and realistic, ideal for medical education, surgical training, and research.^4^ Unlike formalin, which can render cadavers stiff and brittle, Thiel's method employs a unique solution that maintains natural color, texture, and flexibility with reduced toxicity. Widely adopted globally, particularly in Europe, Thiel embalming stands as a preferred choice for its ability to maintain tissue integrity without undue stiffening or distortion although both fresh-frozen and Thiel cadavers serve as valuable models for biomechanical and anatomical research. These cadavers have been effectively applied in training for ultrasound-guided anesthetics, laparoscopic surgery, thyroidectomy, and orthopedic procedures. Additionally, they have more recently been employed for testing medical equipment, including laryngoscopes and joint replacements.^4^ Additionally, they offer longer anatomical stability and a reduced risk of infection compared to fresh-frozen models, ensuring consistency and reliability in biomechanical testing. The realism of Thiel cadavers has been recognized by numerous experts. According to the *World Journal of Surgery*, a survey of 27 surgeons across different specialties found that these cadavers closely resemble live tissue, except for the brain and eyes.^5^ Researchers can replicate clinical scenarios to bridge the gap between theoretical analyses and practical applications.

Screw loosening following locking distal radius plate fixation in orthopedic surgery represents a common complication characterized by displacement beyond allowable.^6^ Although screw pull-out is rare, occurring at a rate of only 0.33% in comprehensive failure cases, screw loosening poses significant challenges.^7,8^ This phenomenon often arises from smaller threads at the screw head, leading to eventual failure due to micromotion.^9^ Interestingly, studies have indicated that the number of screws used in volar locking plates does not significantly impact overall failure rates.^10^ A case verified by Marwan *et al.*^11^ involved a patient with a distal radius fracture in whom screw loosening resulted in a weakened wrist and hand pain alongside numerous complications with nonunion fracture.

Plate fixation stability is influenced by factors, including screw design, bone quality, and loading conditions.^12–14^ Studies by Zhang *et al.*^15^ and Feng *et al.*^16^ highlighted the importance of screw geometry and placement in maximizing fixation strength and minimizing the risk of screw loosening or pull-out. Experimental and finite element analyses of cadaveric radii demonstrated significant correlations between simulated and experimental axial stiffness, underscoring the relevance of considering bone material properties for accurate predictions. While local bone density proved crucial, the impact of local bone anisotropy on predictive abilities was minimal.

In the existing literature, research on screw pull-out from plate fixation has primarily focused on specific scenarios, such as screw design and placement parameters on the fixation strength under pull-out loading.^17,18^ However, there remains a need to explore a wider range of clinical cases to enhance our understanding of screw stability and fixation integrity in en bloc distal radius resection with ulnar reconstruction of distal radius tumors. This study addresses these gaps by integrating finite element analysis (FEA) and experimental investigations on Thiel cadavers to evaluate the screw pull-out behavior from plate fixation in en bloc distal radius resection with ulnar reconstruction.

By subjecting cadaveric specimens to controlled bending loads, we can directly observe screw loosening and pull-out effects, validating FEA predictions and identifying critical fixation parameters. By integrating computational simulations with biomechanical experiments, we seek to gain comprehensive insights into the force limitation for the patients and mechanical responses of the fixation system, providing valuable insights into the biomechanical considerations and surgical implications of locking-plate fixation in complex orthopedic procedures.

## II. RESULTS AND DISCUSSION

### A. Biomechanical testing

**FIG. 1.**
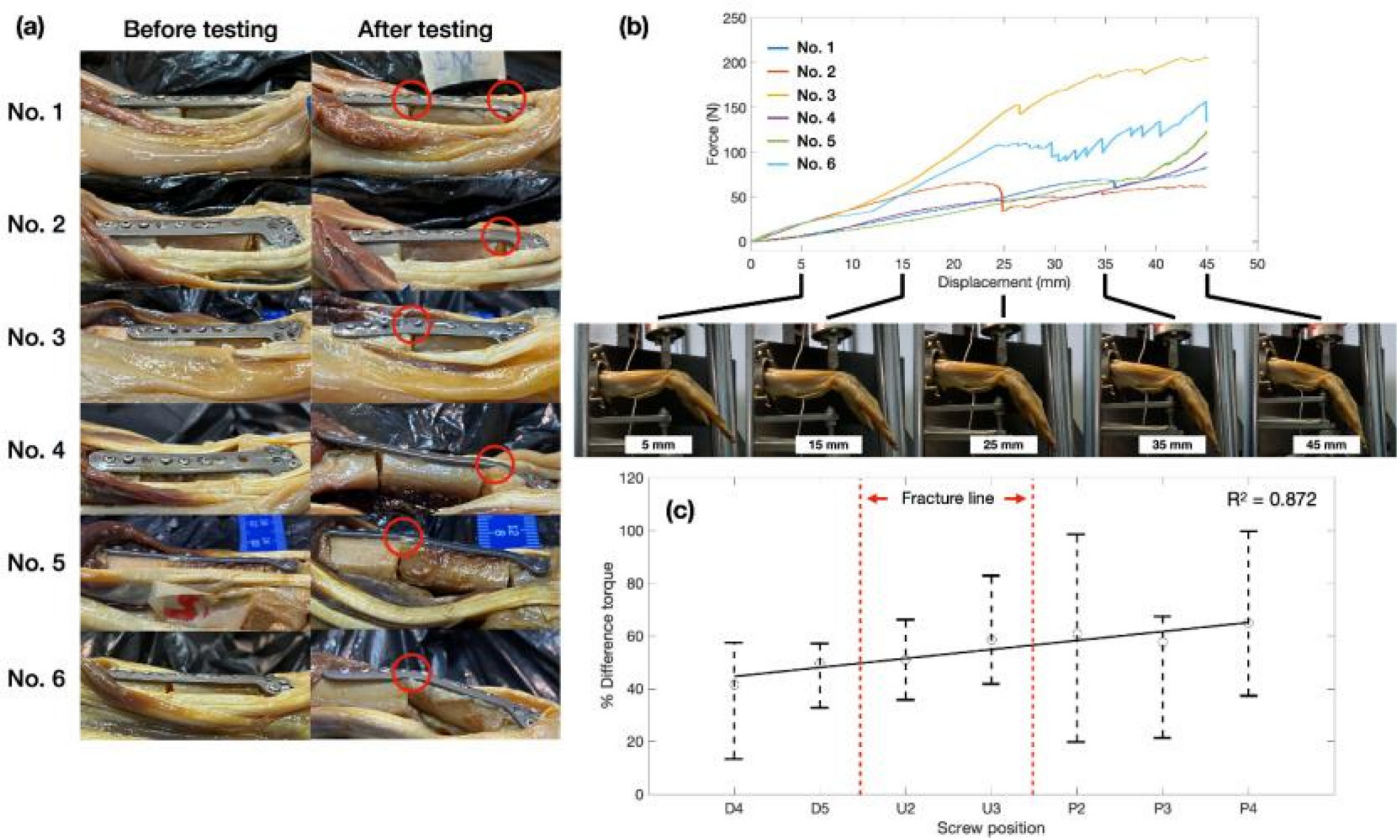
Cadaver testing results. (a) Comparison of screw loosening and deformation of locking plate before and after compression test. The red circles indicate the deformation point of the locking plate. (b) Force and displacement of bending cadaver testing obtained by universal testing machine comparison with physical deformation of specimen no. 3. (c) Percentage difference of screw torque with fracture location (red line) before and after the experiment. The data were extracted from torque measurement as presented in Table I in the Appendix.

**FIG. 2.**
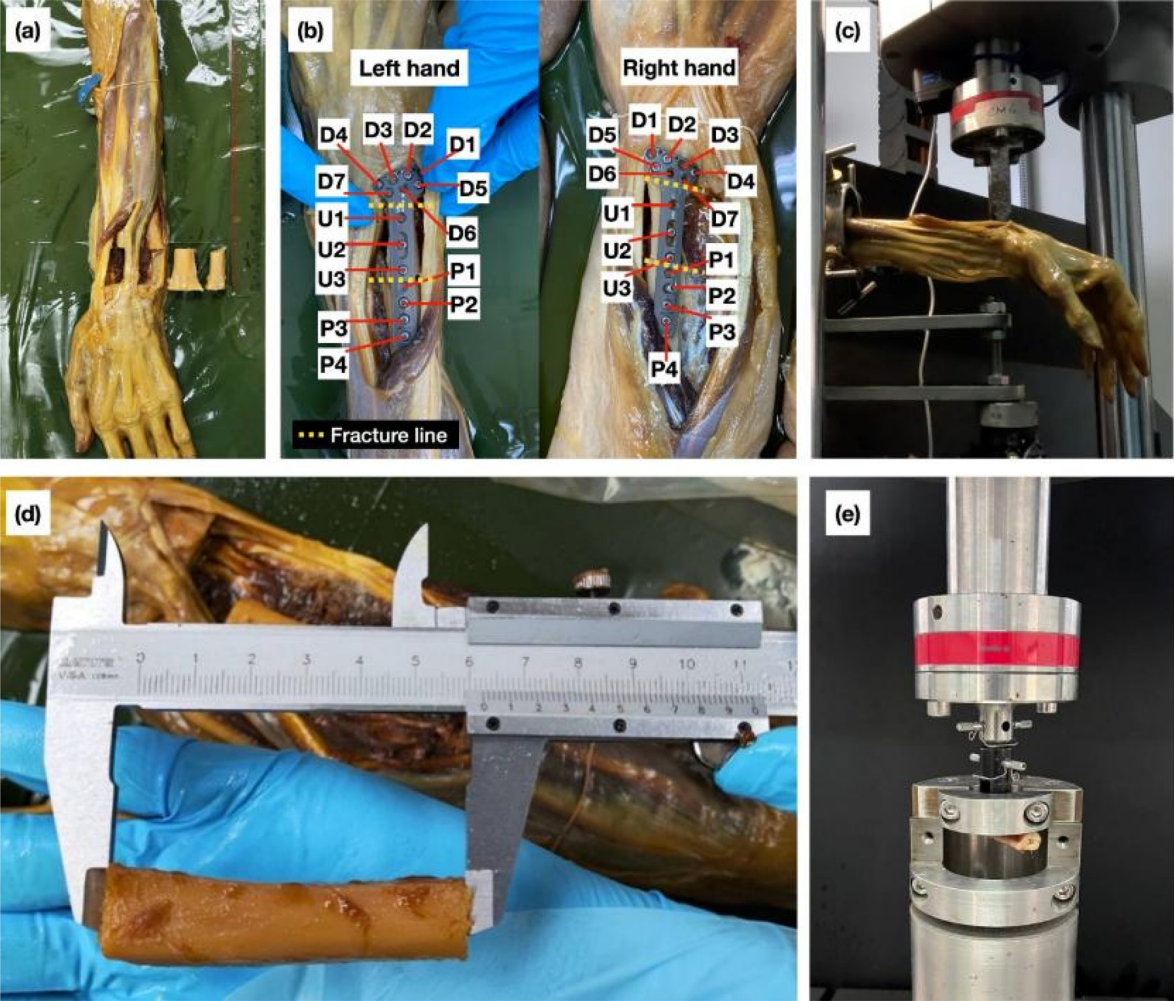
Experimental set-up for en bloc distal radius resection with ulnar reconstruction. (a) Ulnar translocation. (b) Screw configuration and notation with fracture location (yellow line). (c) Experimental set-up for cadaver testing. (d) Preparation of axial screw pull-out testing. (e) Experimental set-up for axial screw pull-out testing. Reproduced with permission from Chancharoen *et al.*, Eng. J. **29**(2), 93–104 (2025). Copyright 2025 Engineering Journal.

To investigate the screw-loosening behaviors, biomechanical testing of the cadaver was performed under cantilever bending conditions. Figure 1 illustrates the results from cadaver testing. As depicted in Fig. 1(a), the deformation of locking plates was compared. The deformation of locking plates was observed between screw positions D5–U1 and U3–P1, as per the notation of screw positioning in Fig. 2(b). Deformation was seen at the location of the cut lines. Figure 1(b) shows the force–displacement relation during the experiment. It was observed that force dropped when the displacement reached 25 mm, causing the initial deformation of the locking plates. However, the experiment continued until the displacement reached the set limit of 45 mm.

The initial tightening torque of each screw secured on the locking plate was measured before and after testing and compared with the value before testing, as reported in Table I of the Appendix. The percentage of difference torque extracted from measured data before and after testing was compared and plotted for each screw, as seen in Fig. 1(c). This could be seen that the initial tightening toque in each screw is 46.05 ± 8.21 N cm. Hence, after performing compression test, it was found that all screws had a torque value lower than torque thresholds (28 N cm). This indicated that screws were slipping. However, screws at proximal location (P2–P4) have a high percentage of torque difference and have a higher chance to be loosened. According to which the screws at the distal zone (D3–D5), where the pressing head was pressed, the screws have low percentage of loosening. This indicates that the screws have a lower chance of coming loose in this area. Otherwise, according to the deformation in Fig. 1(a), regarding the percentage of screw loosening, it was observed that the screws P2 and P4 at the proximal end zone were loose from the bone and locking plates.

**FIG. 3.**
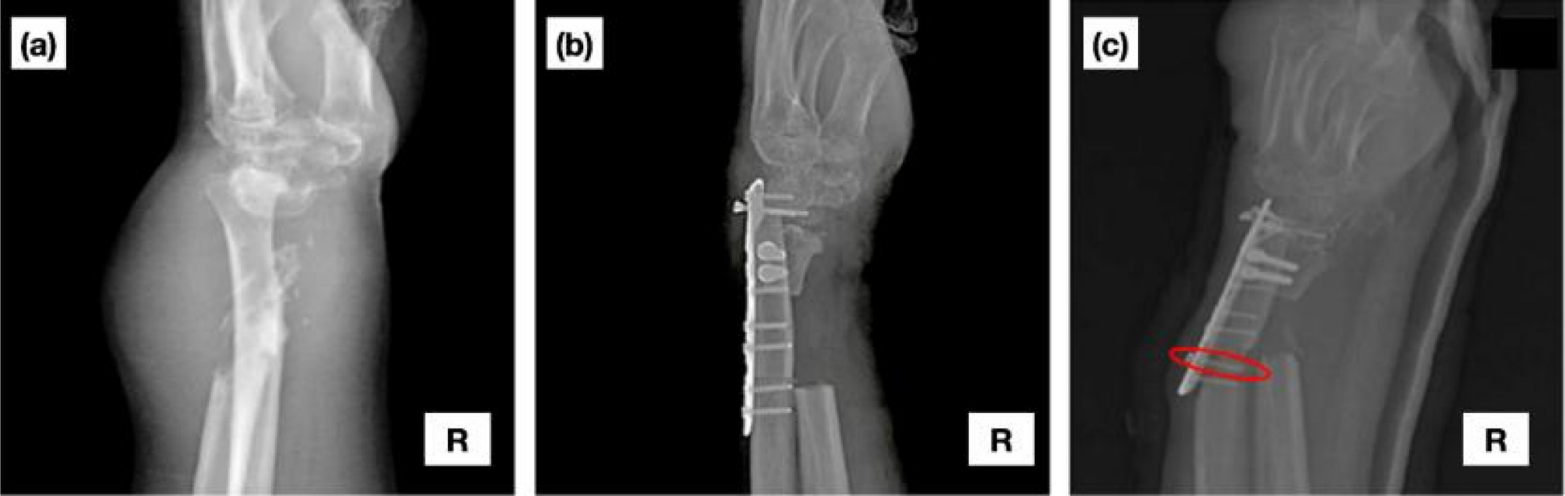
Radiographic comparison of surgical intervention in a 60-year-old man's right hand (a) before and (b) following en bloc resection surgery. (c) Bone deformation and screw loosening from its position at the second bottom of the locking distal radius plate are indicated by a red ellipse.^27^

Moreover, the loosening screw positions were related to the clinical data, as presented in the red ellipse of Fig. 3(c) as well as the comparison of different torque values in Fig. 1(c). Hence, as a result, the difference in torque in each screw could be used to evaluate the chance of screw loosening in clinical conditions. Thus far, from the force–displacement result, the maximum displacement of 45 mm was also applied for the initial input condition in the FEA model.

### B. Screw pull-out force analysis in bone fixation

**FIG. 4.**
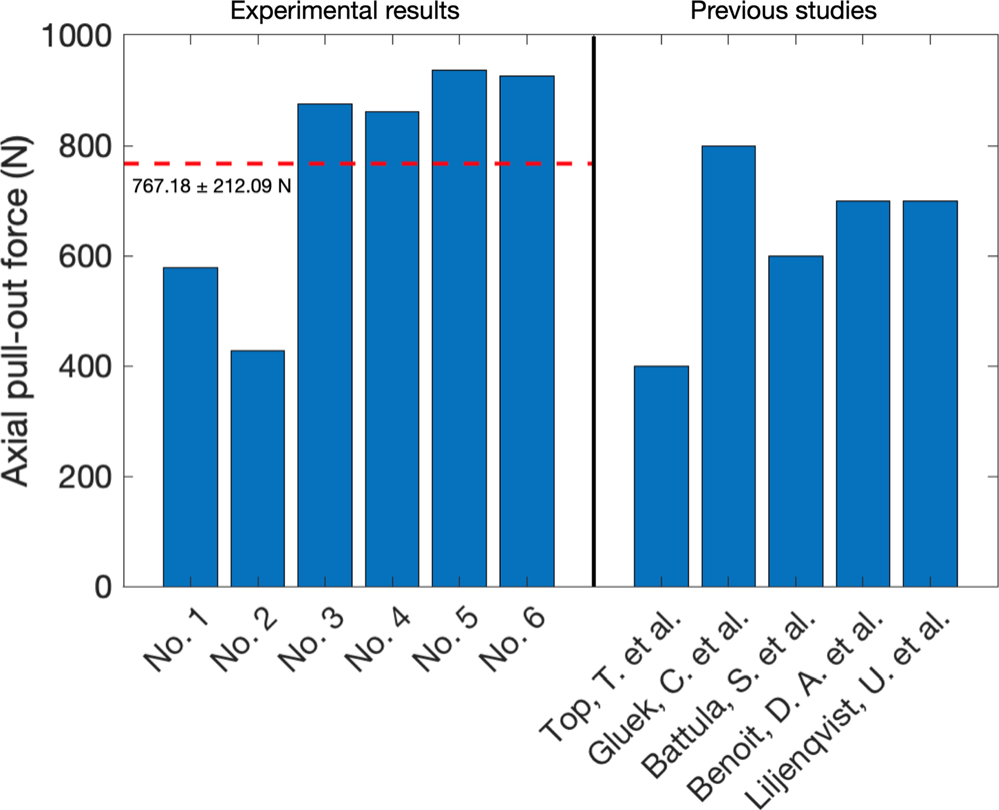
Screw axial pull-out force from six specimens comparing with previous studies. The red line indicates the average with a standard deviation of axial pull-out force from testing results.^19–23^

In conducting screw pull-out force testing, screws were evaluated for their resistance to axial pull-out, replicating the forces encountered within the distal radius bone during stabilization procedures. Figure 4 shows the axial pull-out force of six specimens. The maximum pull-out force observed was 936.17 N in specimen no. 5 before a screw came loose from the bone sample. The average pull-out force of 767.18 ± 212.09 N was also calculated along with the standard deviation to assess data variability. Accordingly, to ensure the effect of screw pull-out force on the variability of bone, the screw pull-out force analysis was compared with previous studies. The range of screw pull-out force was related to the data extracted from previous studies, which fell between 400 and 800 N.^19–23^ However, the varieties of screw pull-out force were depended on test specimens, screw size, as well as cadaver specimens' preparation technique. Even though specimens have a variety of condition, our results were still in the pull-out force range. Thus, this result signifies the screw's ability to withstand considerable tensile forces before dislodgement or failure. Hence, the pull-out force results were used as a criteria range in FEA evaluation for a comparison of biomechanical behavior in each screw.

### C. Finite element analysis and comparisons

#### 1. Stress distribution on locking plate

**FIG. 5.**
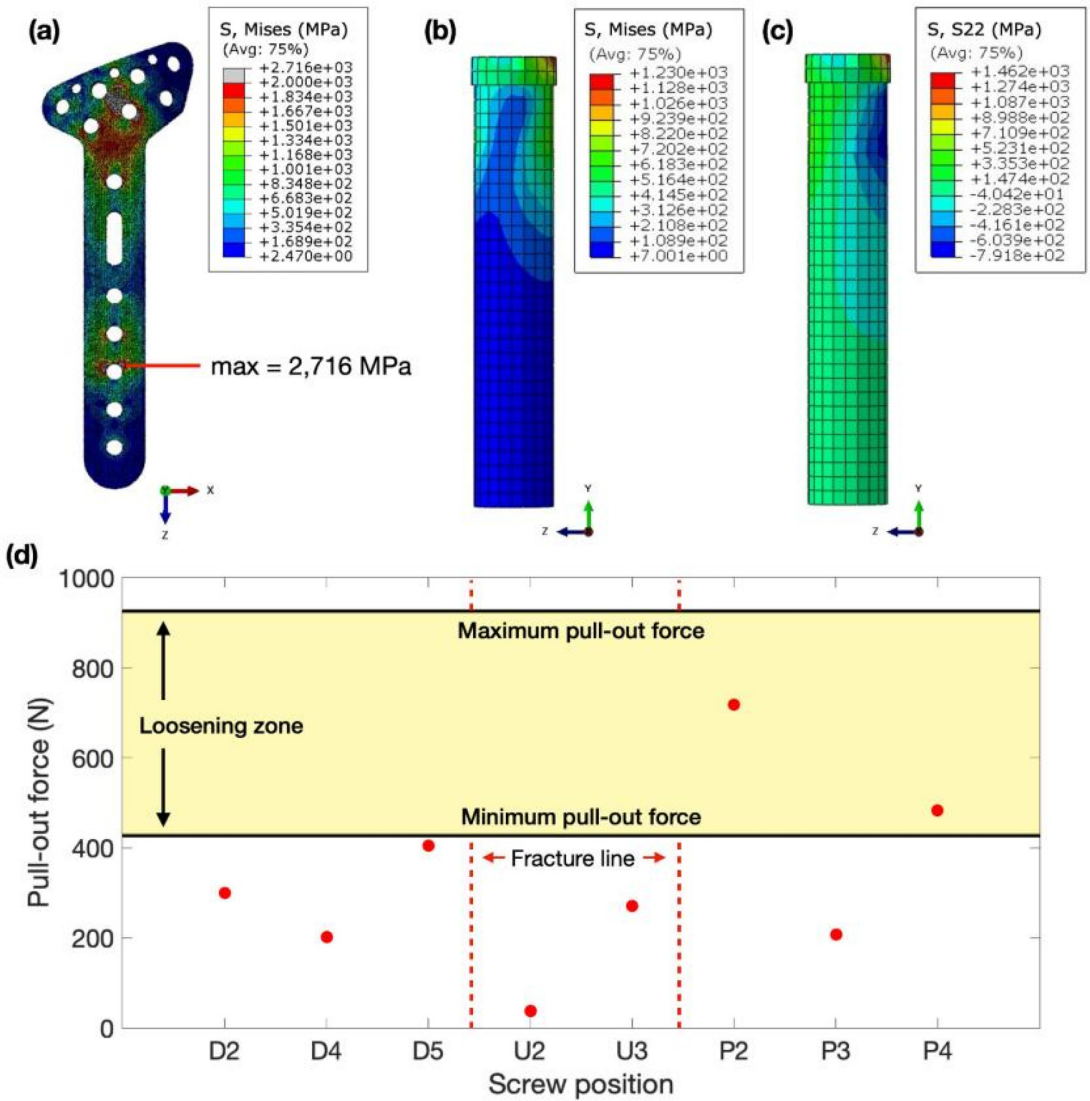
von Mises stress distribution of plate and screw fixation of en bloc distal radius resection with ulnar reconstruction on (a) locking distal radius plate and (b) screw P2. (c) Visualization of stress tensor distribution in screw P2. (d) Comparative analysis of screw pull-out forces depicting results from FEA and the pull-out testing. The red dots, yellow region, and red lines represent the axial pull-out force from FEA, standard deviation of pull-out force extracted from the experiment in Fig. 6 as the screw-loosening zone, and fracture locations on bone, respectively.

The FEA configured all boundary conditions to simulate clinical case scenarios. The von Mises stress within the locking implant plate is concentrated at the interface between pressing head and implant alongside the hole of screw denoted as P2, near the bone gap. At the area of interest of the screw position between P2 and U3, the locking distal radius plate exhibited significant von Mises stress concentrations, with values peaking at 2716 MPa, notably concentrated at screw P2 in close proximity to the bone gap as shown in Fig. 5(a), which illustrates the contour of von Mises stress distribution on the components and the comparison of force extracted from each screw. Comparing simulated stress and deformation outcomes with experimental test results reveals striking similarities in peak stresses and displacements between the two scenarios. This observation suggests promising potential for using simulated results to effectively predict real-world outcomes.

#### 2. Stress distribution and pull-out force on the screw

After conducting the FEA, it was observed that the maximum von Mises stress within the screws for locking-plate fixation reached 1230 MPa. This stress concentration was localized at screw P2, particularly near the bone gap, as depicted in Fig. 5(b). The stress tensor serves as a metric for evaluating failure under applied pull-out forces, as noted by Zdero *et al.*^24^ Another study also aligned with this principle, focusing on identifying a critical stress tensor along the longitudinal axis at the middle of the screw.^25^ Through stress tensor analysis, we determined the maximum stress value of 1462 MPa on screw P2, which is situated closest to the bone gap. This screw experienced the highest stress among all screws, as illustrated in Fig. 5(c). Moreover, the axial pull-out force obtained from FEA could be evaluated by Eq. (1) and plotted as the red dot in Fig. 5(d). According to Fig. 5(d), the range axial pull-out force between 427.71 and 936.17 N from experiment was mapped in order to investigate the position of loosening screw. As a result, the maximum axial pull-out forces obtained by FEA of screws P4 (482 N) and P2 (718 N) fall within the screw-loosening zone identified in Figs. 4 and 5(d). Thus, it could be seen that the screw pull-out force from FEA matched with the compression and screw pull-out experiments. Hence, screw pull-out force analysis from FEA is consistent with the clinical relevance of these findings within the field of orthopedic practice.

According to FEA results, we observed that the screw positions P2 and P4 have chance to be loosened from bone and fixation plate. These results consistent with the compression test. Moreover, when comparing with torque measured after performing compression, this could be observed that screw positions P2 and P4 have the higher in percentage difference. These results were matched with the high stress tensor and screw pull-out force from FEA. Nonetheless, the torque results could not be directly compared with the screw pull-out force value. The percentage difference could be used to anticipate the location of loosened screw, while screw pull-out force was used to determine the value that causes the screws to be loosened.

## III. CONCLUSIONS

In this study, we investigated the mechanism of screw pull-out forces in plate fixation for en bloc distal radius resection with ulnar reconstruction. Using FEA and experimental data from Thiel cadavers, we examined pull-out force along screw axes. The significant axial pull-out force observed in the screw situated nearest to the bone gap further validated this investigation's clinical relevance. Comparing screw pull-out forces between FEA and biomechanical tests revealed alignment, particularly noting that the screw close to the bone gap and the screw at the first bottom of the locking distal radius plate fell within the screw-loosening zone identified in biomechanical tests. Understanding critical screw locations and the screw-loosening zones is essential for surgical planning and execution. This study emphasizes the importance of screw placement, particularly near the bone gaps, to minimize the risk of complications. Clinically, our study emphasizes the importance of placing more screws in the near bone gaps area to resist bending moments, thus improve fixation stability. Additionally, the employment of longer screws in distal fragment fixation may enable resistance against pull-out failure, especially for high stress situations.

In summary, this research provides insights into the biomechanical aspects of screw pull-out in complex orthopedic procedures. By integrating computational and experimental methodologies, we offer practical implications for surgical strategies and patient outcomes in distal radius resection with ulnar reconstruction.

## IV. METHODS

**FIG. 6.**
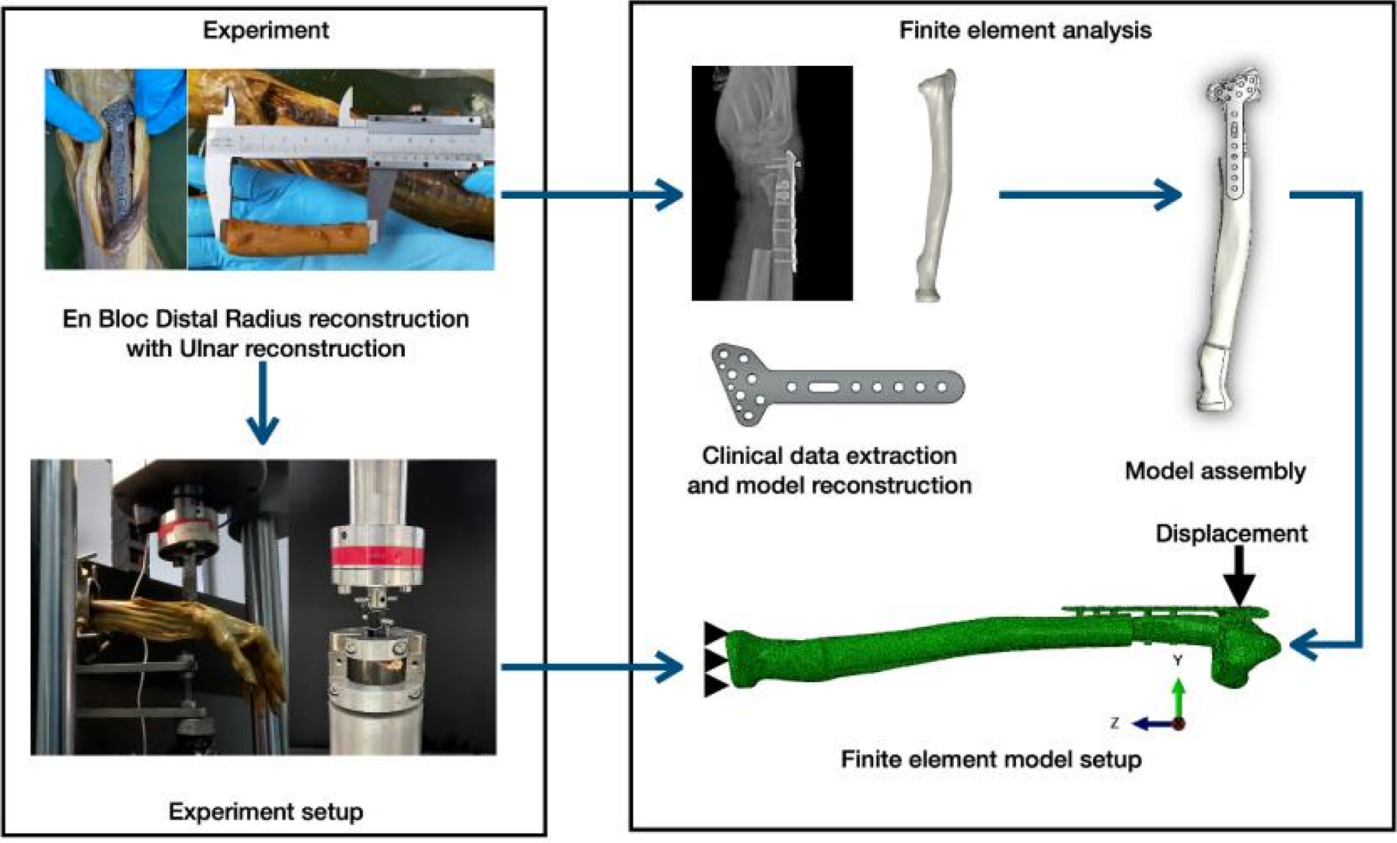
Workflow of experimental and computational analyses for evaluating screw pull-out force from plate fixation in en bloc distal radius resection with ulnar reconstruction.

Figure 6 illustrates the workflow for evaluating screw pull-out from plate fixation in en bloc distal radius resection with ulnar reconstruction. The research integrates experimental and computational methodologies to comprehensively evaluate the phenomenon. The methodology encompasses various stages, including clinical data extraction, experimental set-up, computational analysis, and validation of results, thus ensuring a thorough investigation into screw pull-out from plate fixation in en bloc distal radius resection with ulnar reconstruction.

### A. Clinical data extraction

Figure 3 shows the clinical scenario under investigation involving the en bloc resection of a segment of the distal radius in a 60-year-old male patient, with resection 4 cm in length.^26^ Following tumor removal, reconstructive surgery employed ulnar translocation using a vascularized graft to replace the excised portion of the distal radius [Fig. 3(a)]. Notably, the scapholunate region was incised to accommodate the vascularized ulnar graft, which was subsequently secured using a locking distal radius plate in conjunction with cortical screws, as depicted in Fig. 3(b). Subsequent radiographic examination 1 month after surgery revealed significant deformation of the vascularized ulnar graft along with loosening of a screw positioned at the second bottom of the locking distal radius plate, as highlighted in Fig. 3(c). The surgeon attributed the screw loosening to the patient's habit of lifting objects, resulting in its displacement over the course of the month and necessitating further surgical intervention.^27^ Figure 3 provides information regarding the causal relationship underlying the patient's case, prompting consideration for pre-operative biomechanical assessment to mitigate such complications.

### B. Preparation of cadaver specimens

Six limbs were obtained from three donors aged 66–88 years at death from the Department of Anatomy, Faculty of Science, Mahidol University in Bangkok, Thailand. Two were males, and one was female. They had pre-arranged the donation of their bodies to the department with informed consent obtained before their death. The bodies were embalmed using Thiel's method. Thiel-embalmed cadavers were employed due to their superior soft tissue preservation, which is a more realistic surgical environment and implant placement compared to formalin-fixed specimens. Compared to fresh-frozen cadavers that develop tissue degeneration over time, Thiel cadavers have anatomical stability, reducing variation in biomechanical studies. Furthermore, ethical and procedural constraints in our department made it impossible for us to employ fresh cadavers in this experiment.

While another study by Stefen *et al.*^28^ demonstrated that Thiel embalming could alter bone biomechanics, our interest is in relative mechanical performance of the implant rather than absolute bone strength. The Thiel cadavers provide a reasonable compromise between preservation of soft tissue integrity and the potential for biomechanical testing so that our findings can still be compared to clinical reality. The embalming process and chemicals used followed the protocol outlined by Eisma *et al.*^4^ In brief, the cadavers were injected with two solutions: arterial and venous. The arterial solution was perfused through the femoral artery, while the venous solution was perfused into the superior sagittal sinus. Thereafter, the cadavers were submerged in tank fluid for 4–6 months before being used in this study. The specimens were kept moist within sealed wraps to reduce exposure to air and prevent excessive dehydration of the specimens under room-temperature conditions.

Figure 2 illustrates the sample preparation and experimental set-up of the cadaver specimens. To simulate the en bloc resection of the distal radius, in the referenced clinical case, the ulna and the radius were cut, as shown in Fig. 2(a), following the orthopedic surgeon's supervision and advice. Two cuts were made on the radius. The first cut was positioned 1 cm proximally away from the radiocarpal joint line, and the second was made 3.5–4 cm proximally away from the first cut. The same cuts were then made on the ulna. During the incision process for the en bloc resection, utmost care was taken to preserve the major ligaments and soft tissues on the specimens.

The translocation process then proceeded by replacing the prepared radius section with the prepared ulna. The fixation process was done by following standard procedures. Pilot holes were drilled into the sections and then fixated using dorsal distal radius plate implants (Stryker dorsal smartlock DR plate standard extra-long; Stryker, Kalamazoo, MI, USA). The plates were then secured by using the screw configuration of the clinical case,^27^ with three 2.3-mm locking screws (T7 with a diameter of 2.3 mm and 12-mm-long locking screws; Stryker) at the distalmost region (D2, D4, and D5) and five 2.7-mm locking screws (T7 with diameter of 2.7 mm and 16-mm-long locking screws; Stryker) on the ulnar (U2 and U3) and proximal (P2–P4) section of the plates. The location of screws and configurations were denoted as illustrated in Fig. 2(b).

### C. Cantilever bending test

After the specimens were prepared, the testing was conducted with the Instron ElectroPuls E10000 universal testing machine (Instron, Norwood, MA, USA) at the Research Instrument Center, Khon Kaen University, Thailand. The testing set-up is shown in Fig. 2(c). The prepared forearms were secured with the mounting fixture, with the screwed-on locking mechanism screwing onto the bones of the forearms to ensure that the specimens were stable throughout the testing. The distance between the center of the sets of securing screws nearest to the center of the press head of the machine was 15.6 cm in all tested specimens to fix the distance of bending moment. The pressing head was placed at the distalmost region of the radius, slightly overlapping the installed dorsal locking plates. The loading set-up aims to simulate the action of lifting heavy loads when pointing the arm forward with the palm side down. The compression rate was set to 2 mm/min, with a displacement limit of 45 mm.

To ensure that the screw is in threshold torque (28 N cm) and not exceed the failure torque (above 70 N cm), the initial tightening torque of the screws was measured by using an electronic torque screwdriver (ES.404 Electronic Torque Screwdriver; FACOM, Morangis, France).^29^ The initial tightness of the screws during the fixation process was in accordance with the supervising surgeon's advice. Therefore, the torque after performing compression test was measured to anticipate the screw-loosening location. The results of torque in each screw are presented in the Appendix.

### D. Screw pull-out test

In this study, the screw pull-out testing follows the standard ASTM F543 procedures to obtain the pull-out strength.^30^ Six Thiel-cadaveric radius bone specimens were collected from the same corresponding Thiel-cadaveric forearms mentioned in Sec. IV B. The cut specimens were approximately 6 cm in length, as shown in Fig. 2(d). After the cut radius specimens were obtained, the same procedures described in Sec. IV B were repeated to implant the screws into the specimens. 2.7-mm locking screws (T7 with a diameter of 2.7-mm locking screws; Stryker) were installed onto the cut radius specimens. Following the ASTM F543 A2 guidelines, the screws were installed such that 60% of the threaded region of the screws was embedded inside the specimens.

The screw pull-out force testing was conducted to measure the range of force that causes the loosening of screws with the Instron ElectroPuls E10000 universal testing machine at the Research Instrument Center, Khon Kaen University, Thailand. The testing set-up is shown in Fig. 2(e). The prepared specimens' screw heads were secured onto the load fixture, and then the “bone” section of the specimen was secured onto the test bed (load cell side) using the grip clamp to secure the specimen in place when the pull-out load was applied. The pull-out testing displacement rate was set to 2 mm/min to replicate the cantilever loading case described in Sec. IV C.

### E. Finite element modeling and simulation

#### 1. Model development and material properties

**FIG. 7.**
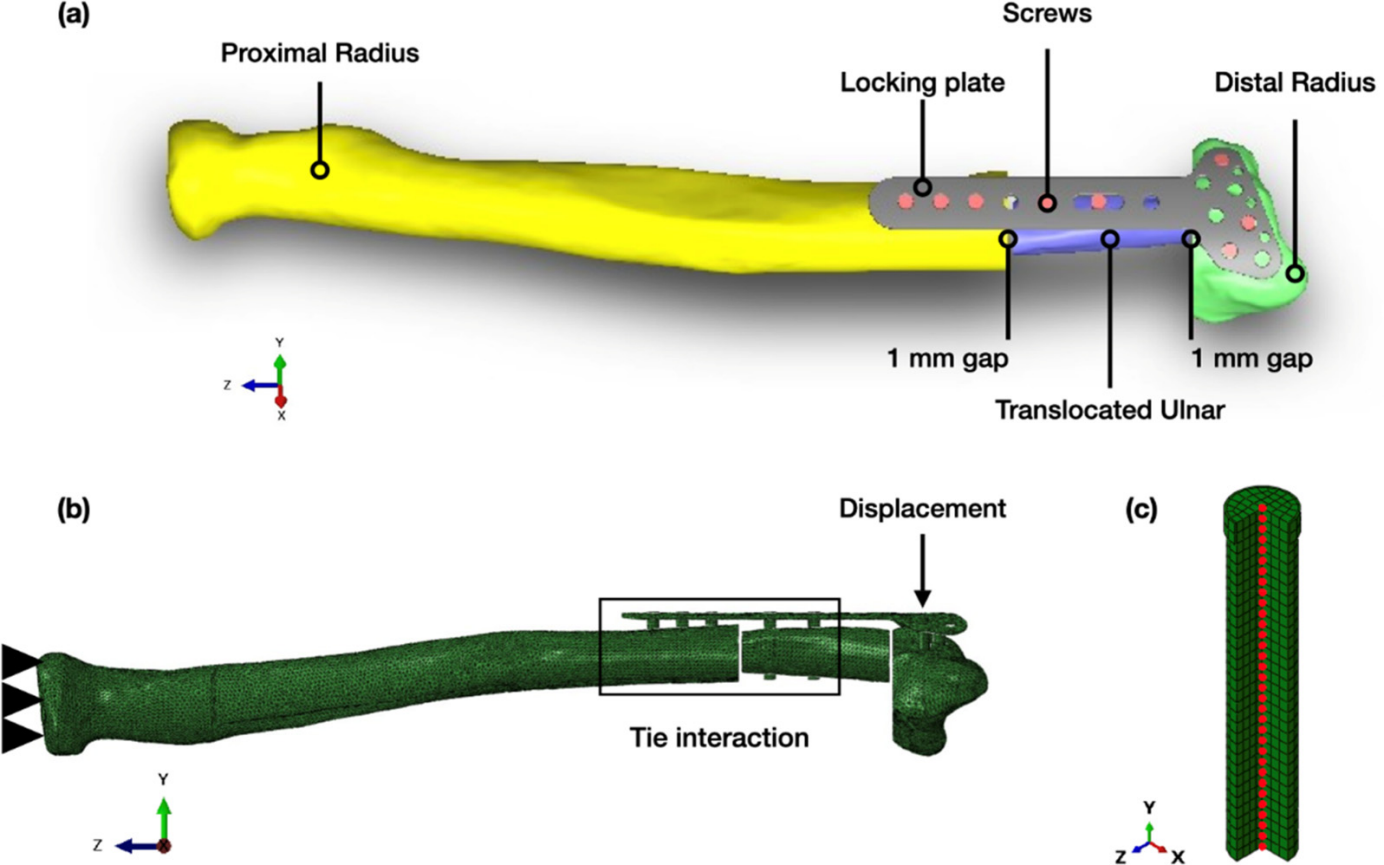
Schematic of the three-dimensional (3D) assembled model. (a) 3D assembled finite element model with bones, locking plate, and screws. (b) Finite element model with boundary conditions and interactions. (c) Finite element model of screw meshing and half-section view illustrating the core of the middle node of the screw.

FEA using ABAQUS/CAE was employed to illustrate the mechanical behavior of the plate fixation system in en bloc distal radius resection with ulnar reconstruction. The geometry of the ulnar and distal radius bones was obtained using computed tomography scans, converted to DICOM format for segmentation and subsequently reconstructed into STL format for further manipulation and analysis. Next, simplified cylindrical models with a diameter of 2.7 mm and a length of 16 mm, and locking distal radius plates, were employed to analyze screw pull-out behavior accurately in SOLIDWORKS (Dassault Systèmes, Vélizy-Villacoublay, France). The models were assembled based on a 1-mm bone gap between the radius and ulna on both sides to replicate the clinical case's resection length, as depicted in Fig. 7(a).

The material properties of the bone, screws, and implant materials were accurately defined based on empirical data. The material properties of radius, ulna, scaphoid, and lunate bones were assumed homogeneous, isotropic, and elastic, adhering to standard biomechanical modeling assumptions discussed in the literature.^31^ Recent studies have demonstrated that assuming bone as a homogeneous material properties does not significantly affect stress distribution in certain finite element models, although cortical and cancellous bone exhibit anisotropic behavior.^32,33^ Additionally, ligaments, muscles, and tendons were not included in the model, as their omission has been shown to have a minimal impact on the specific loading conditions analyzed in this study.^34^ Moreover, the cancellous bone contributes less to screw fixation strength in bending-dominated conditions, and this limitation has a minimal impact on the overall conclusions of this study. Hence, Young's modulus value for bone of approximately 14.4 GPa and a Poisson's ratio of 0.3 were adopted in this study. This value was derived through a comprehensive review of the literature, including studies by Mancuso *et al.*, Liu *et al.*, and Pramudita *et al.*^35–37^ The screws and locking-plate implant were modeled using titanium alloy Ti-6Al-4V with a Young's modulus of 113.8 GPa and a Poisson's ratio of 0.34, a standard material property in orthopedic implants.^38,39^

#### 2. Interaction, meshing, and boundary conditions

The interaction between the screws and the plate implant was simulated using the tie interaction method,^40^ preventing relative movement between the plate and screws. The same constraints were applied to the screws and bones because the screws showed no pull-out displacement in testing. The radius and ulnar bones were modeled with tie constraints to simulate a fully bonded situation with surrounding tissues, as depicted in Fig. 7(b).

Load cases representing physiological and worst-case scenarios were applied to the model. To replicate the experimental set-up, the proximal end of the radius bone was rigidly fixed, preventing translational or rotational movement. Additionally, to simulate physiological wrist mechanics, a displacement of 45 mm was applied to the head of the locking distal plate implant, corresponding to the limited displacement in the experiment described in Sec. IV C. The model set-up is illustrated in Fig. 7(b).

In this study, distinct meshing techniques were tailored to the structural characteristics of bone and implant components. Quadratic tetrahedral elements (C3D10) were used for bones and implants, effectively modeling complex geometries for biomechanical analysis. Meanwhile, hexahedral elements (C3D8) were employed for screw components, capturing efficiently the mechanical behavior of solid structures. Hence, mesh sensitivity analysis was performed by systematically refining the mesh and evaluating the convergence of key mechanical parameters of interest.

#### 3. Screw pull-out force analysis

To comprehensively evaluate screw pull-out behavior, the FEA technique complemented by experimental investigations on Thiel-cadaveric specimens was employed. A detailed examination of stress distribution within the screws was conducted using stress tensor analysis. This method enabled precise identification of critical stress concentrations, facilitating practical measures to mitigate potential failure modes.

The insight provided by Zdero *et al.*^24^ and Chancharoen *et al.*^25^ was applied in this study, which highlighted that the highest stress leading to pull-out failure typically occurs along the longitudinal axis of the screw. Therefore, the stress tensor was employed to measure failure resulting from a pull-out force on the screw. Radial meshing was performed, focusing on the calculation of pure axial pull-out force to avoid screw-bending stress. The analysis concentrated on middle nodes, which were unaffected by bending stress, enabling evaluation of axial pull-out force as shown in Fig. 7(c). The axial force on each screw was calculated by multiplying stress at the middle node by the cross-sectional area, yielding the pull-out force parameter.^24,25^ To calculate the axial pull-out force *F*_axial_ on the screw due to pull-out (N), an equation is used,

$$F_{\text{axial}}=A_{\text{thread}}\times \sigma_{\text{max}},$$ (1)

where *A*_thread_ represents the cross-sectional area (mm^2^) of the screws, which calculated from screw specification including thread diameter, and *σ*_max_ denotes the maximum axial stress in Y direction (MPa) within the screw obtained by FEA. This comprehensive approach provides a thorough estimation of the pull-out force exerted on the screw during pull-out scenarios.

In addition to stress tensor analysis, deformation analysis of the en bloc distal radius resection with ulnar reconstruction based on fundamental principles of mechanics was explored. It was determined that after surgery, the allowable displacement of the distal radius should not exceed 3 mm, consistent with established clinical standards and previous research findings.^41^

## Data Availability

Data sharing is not applicable to this article as no new data were created or analyzed in this study.

## APPENDIX: MEASUREMENT OF TORQUE ON LOCKING SCREWS

As mentioned in the cantilever bending test section and Fig. 1(c), torque on locking screws was measured before and after performing a cantilever bending test for cadaver specimens. The torque on each screw was compared to analyze the percentage of screw loosening, as presented in Table I.

**TABLE I.** Torque of locking screws at each location referred to in Fig. 3(b).

		Torque (cN m)						
		Screw position						
	Sample No.	D4	D5	U2	U3	P2	P3	P4
1	Before	40.33	49.80	57.50	76.73	61.63	58.90	55.23
	After	34.80	33.40	27.40	43.90	49.30	46.10	34.50
	%Difference	13.72	32.93	52.35	42.79	20.01	21.73	37.54
2	Before	45.43	43.60	40.97	45.53	53.37	52.63	50.20
	After	20.30	24.30	26.10	26.40	22.30	21.80	25.40
	%Difference	55.32	44.27	36.29	42.02	58.21	58.58	49.40
3	Before	34.50	44.27	31.07	39.90	0.00	39.63	43.57
	After	21.20	20.60	17.60	6.70	0.00	15.30	12.60
	%Difference	38.55	53.46	43.35	83.21	0.00	61.40	71.08
4	Before	35.03	48.20	47.40	44.23	45.13	35.13	41.83
	After	23.20	19.80	21.40	21.10	15.60	11.50	0.00
	%Difference	33.78	58.92	54.85	52.30	65.44	67.27	100.00
5	Before	45.67	42.70	53.70	46.37	40.57	43.67	42.63
	After	19.50	20.60	17.50	18.30	14.50	13.20	13.70
	%Difference	57.30	51.76	67.41	60.53	64.26	69.77	67.87
6	Before	45.20	42.63	45.20	48.33	42.00	43.37	42.83
	After	21.80	18.50	21.40	14.20	18.00	14.40	15.10
	%Difference	51.77	56.61	52.65	70.62	57.14	66.79	64.75
